# Diverse Wild Bird Host Range of *Mycoplasma gallisepticum* in Eastern North America

**DOI:** 10.1371/journal.pone.0103553

**Published:** 2014-07-25

**Authors:** André A. Dhondt, Jonathan C. DeCoste, David H. Ley, Wesley M. Hochachka

**Affiliations:** 1 Bird Population Studies, Laboratory of Ornithology, Cornell University, Ithaca, New York, United States of America; 2 Department of Population Health and Pathobiology, College of Veterinary Medicine, North Carolina State University, Raleigh, North Carolina, United States of America; Arizona State University, United States of America

## Abstract

Emerging infectious diseases often result from pathogens jumping to novel hosts. Identifying possibilities and constraints on host transfer is therefore an important facet of research in disease ecology. Host transfers can be studied for the bacterium *Mycoplasma gallisepticum*, predominantly a pathogen of poultry until its 1994 appearance and subsequent epidemic spread in a wild songbird, the house finch *Haemorhous mexicanus* and some other wild birds. We screened a broad range of potential host species for evidence of infection by *M. gallisepticum* in order to answer 3 questions: (1) is there a host phylogenetic constraint on the likelihood of host infection (house finches compared to other bird species); (2) does opportunity for close proximity (visiting bird feeders) increase the likelihood of a potential host being infected; and (3) is there seasonal variation in opportunity for host jumping (winter resident versus summer resident species). We tested for pathogen exposure both by using PCR to test for the presence of *M. gallisepticum* DNA and by rapid plate agglutination to test for the presence of antibodies. We examined 1,941 individual birds of 53 species from 19 avian families. In 27 species (15 families) there was evidence for exposure with *M. gallisepticum* although conjunctivitis was very rare in non-finches. There was no difference in detection rate between summer and winter residents, nor between feeder birds and species that do not come to feeders. Evidence of *M. gallisepticum* infection was found in all species for which at least 20 individuals had been sampled. Combining the present results with those of previous studies shows that a diverse range of wild bird species may carry or have been exposed to *M. gallisepticum* in the USA as well as in Europe and Asia.

## Introduction

*Mycoplasma gallisepticum* conjunctivitis emerged in house finches *Haemorhous mexicanus* during the winter of 1993-94 in Maryland, USA [1], [2], became a large-scale epidemic [3]–[8], and is now endemic throughout most of the house finches' North American range. Although a well-documented bacterial pathogen of poultry world-wide, this marked the first epidemic of *M. gallisepticum* in wild birds [9]. Phylogenetic evidence shows a single origin for the epidemic and rapid evolutionary changes of the pathogen as it expanded geographically [7], [10]–[13] and in host range over time[10], [12], [14], [15]. The ongoing continent-wide epidemic in house finches and a local epidemic in Quebec in pine grosbeak *Pinicola enucleator* and evening grosbeak *Coccothraustes vespertinus* in 1998-99 was the result of a single successful host jump [11], [16]. Two independent coalescence analyses based on largely independent sets of isolates of *M. gallisepticum* suggest that this lineage of bacteria in house finches diverged from the *M. gallisepticum* found in poultry a few years prior to the start of the recognized conjunctivitis epidemic in house finches [12], [13]. In the time between the initial divergence of “house finch” *M. gallisepticum* and the time of disease outbreak, the bacteria may have been evolving within this single new host.

Since 1994 largely anecdotal evidence has accumulated showing that a larger number of bird species may be infected by *M. gallisepticum*, and hence potentially be involved in the population-level host-disease dynamics of *M. gallisepticum* in wild songbirds. A number of bird species potentially interact with and could be exposed to *M. gallisepticum* from house finches, particularly because these species come into close proximity at bird feeders. To date, *M. gallisepticum* has been isolated in North America from seven free-ranging species, including five fringillid finches, and 18 species have been detected carrying antibodies against *M. gallisepticum* [17]–[19] (Table S1 and [16]). Although conjunctivitis can have various causes it is interesting to mention that visual observations at bird feeders suggest an even greater range of possible wild bird hosts for *M. gallisepticum*, based on 675 reported cases of conjunctivitis in 31 species other than house finches reported by 3489 citizen scientists across 37 states and six Canadian provinces [20]. All of these additional species could have been exposed to *M. gallisepticum* via infected house finches, but an alternative explanation for the diversity of hosts may be that *M. gallisepticum* has been present among wild birds undetected for an extended period. Unappreciated wild bird reservoirs are possible, although these might also be introductions from poultry reservoirs. For example, in 2001 we isolated *M. gallisepticum* from an Ithaca, NY house finch. Phylogenetic analysis showed that this isolate had a poultry association, and was not part of the house finch clade, which suggests introduction of a strain which was not sustained in house finches [11]. Lending credence to the possibility that *M. gallisepticum* is routinely circulating in wild birds and/or introduced from known reservoirs are observations of *M. gallisepticum* in wild birds outside of North America and before the North American epidemic emerged. The few studies that have tested for the presence of *M. gallisepticum* in free-living birds in other parts of the world found evidence for its presence in wild house sparrows *Passer domesticus* in India [21], wild tree sparrows *Passer montanus* in Japan [22] and more recently in various corvid species in Scotland [23], [24] and Malaysia [25]. Potentially, *M. gallisepticum* could be widespread geographically and occur, usually without causing obvious signs of disease, in a wide variety of host species. The extent and importance of infection of hosts other than house finches is poorly known. Systematic and intensive sampling of wild bird species for the presence of *M. gallisepticum* and subsequent phylogenetic placement is necessary to identify the true prevalence and epidemiology of this bacterium outside of its typical galliform hosts.

The objective of this study was to survey a large a proportion of a local bird community for evidence of *M. gallisepticum*, and to identify patterns in the variation of exposure prevalence. We conducted this work around Ithaca, NY, a region in which *M. gallisepticum* is endemic in house finches and in which we would expect the potential for transfer of *M. gallisepticum* among host species. The potential for transfer should be present particularly for species associated with bird feeders and with species that occur in this area during the fall and winter when disease prevalence is highest in house finches [26]. Using both PCR and antibody diagnostic tests we identified both current and past infections of the birds that we screened in order to determine if *M. gallisepticum* (1) is more prevalent in house finches than in other bird species;(2) is more prevalent in fringillids compared to other bird families; (3) is more prevalent in birds associated with feeders than in birds that rarely use feeders; (4) is more frequent in winter than in summer visitors; and (5) prevalence estimates differs between detection methods.

## Materials and Methods

### Ethics Statement

Wild birds were trapped using mist nets and cage traps under New York State Fish and Wildlife License 39 (Albany, NY) and permit 22669 from the United States Geological Survey, Department of the Interior (Laurel, MD). All sampling procedures were approved by Cornell University's Institutional Animal Care and Use Committee (permit 2006-094).

### Study of wild birds

Wild birds were studied between January 2007 and June 2010 in Tompkins County, New York (42°46′ N, 76° 45′ W). At several locations in Tompkins County we maintained bird-feeding stations throughout the year. Feeders were baited with black-oil sunflower seeds. Trapped birds were banded with a unique aluminum leg band (USGS) at the time of initial capture. Eyes were scored for gross lesions on a 0 (no visible lesions) to 3 (severe lesions) scale [27]. After examination, conjunctival samples were collected from each eye using separate polyester tipped aluminum swabs (Puritan Medical, Guilford, ME) to inoculate one tube of Frey's mycoplasma broth medium with 15% swine serum (FMS) thus creating a pooled sample for each bird for mycoplasma culture and isolation [28]. Additionally, a blood sample was taken from the brachial vein into a heparinized capillary tube and immediately put on ice. Plasma was separated from blood and within 24 hours of sampling tested for *M. gallisepticum* antibodies by rapid plate agglutination (RPA) using commercially available *M. gallisepticum* antigen (Charles River Laboratories, Inc). All tests included positive and negative controls. A positive result indicates exposure to *M. gallisepticum* sufficient to induce an immune response producing detectable circulating antibodies [29]. In house finches *M. gallisepticum* antibodies can still be found more than a year after recovery from an experimental infection [27] although antibodies may become undetectable within weeks after infection [30]. A negative RPA result, therefore, does not mean a bird has never been exposed to *M. gallisepticum*.

To determine the presence of current infections pooled conjunctival swab samples from 2007-2009 were tested directly for *M. gallisepticum* by polymerase chain reaction (PCR). A positive result indicates the presence of *M. gallisepticum* DNA in the sample and most likely a current infection. In order to increase the probability of successful culture the 2009–2010 samples were placed in FMS and incubated at 37°C for one week to enrich for mycoplasmas [31], after which a 25 µl aliquot was divided into three samples each of which was tested for the presence of *M. gallisepticum* DNA using conventional PCR [32]. The remaining part of each incubated sample was then stored at −70C. At the outset, samples were tested for *M. gallisepticum* DNA using 16S rRNA gene primers [33]. Starting in 2009, samples that were *M. gallisepticum* positive with the 16S rRNA primers were also tested using *mgc*2 primers [34]. Prior to 2009, frozen samples positive with16S rRNA gene primers were shipped on dry ice to David H. Ley at North Carolina State University, College of Veterinary Medicine, Raleigh, NC for mycoplasma culture, isolation, and identification. Finding limited culture success with 16S-positive samples, starting in 2009 we only sent *mgc2-*positive samples for mycoplasma culture, and conjunctival swabs were inoculated into BD/Copan Universal Transport Medium (UTM) (Becton, Dickinson and Company, Sparks, MD) instead of FMS and stored at −70° to enhance the possibility of successful mycoplasma culture from PCR-positive samples (see below).

For data analyses birds were grouped in various ways to explore possible effects of feeder use, migratory status, and taxonomy on *M. gallisepticum* exposure. Although the total sample size seems large, we do not have enough data to correct for seasonal effects in our comparisons of percentages of individuals that tested positive. Comparisons of percentages between groups were calculated using the distribution-free Mann-Whitney U test (Statistix 8.2, Analytical Software, Tallahassee, Florida). The two-tailed P-value of the result is reported based on the exact permutation test corrected for ties. Means are reported with standard errors (SE). Frequencies were compared using a Chi-square test.

## Results

### Range of hosts

A large number of bird species tested positive for exposure to *M. gallisepticum*. Among the 1,941 individuals of 53 species sampled between 2007 and 2010, individuals of 11 species were positive for *M. gallisepticum* both by PCR and RPA, and 27 of 53 species belonging to 19 avian families were positive either by RPA and/or by PCR (Table 1). The probability of at least one positive test for a species increased rapidly with sample size: using both methods *M. gallisepticum* was detected in only four (16%) of 25 species with samples of five individuals or less. In this group only one species (wood thrush *Hylocichla mustelina*) was positive with both tests. Among the 13 species for which we sampled between six and 19 individuals, eight species (62%) had at least one positive test, although none were positive by both tests. In all 15 species for which we trapped at least 20 individuals evidence for exposure to *M. gallisepticum* was found (Table 1). In this group 10 species (67%) were positive by both tests (though not necessarily the same individual), two were positive by PCR only (red-winged blackbird *Agelaius phoeniceus*, purple finch *Haemorhous purpureus*), and three were positive by RPA only (tufted tit *Baeolophus bicolor*, black-capped chickadee *Poecile atricapillus*, gray catbird *Dumetella carolinensis*). Conjunctivitis, typical of *M. gallisepticum* infection, was observed in four species: house finch (9.4% of total samples n = 331), American goldfinch *Spinus tristis* (0.7% of n = 537), purple finch (3.6% of n = 28), and black-capped chickadee (0.6% of n = 160). Only in the two species with the largest sample sizes (house finch, n = 331; American goldfinch n = 537) were all three criteria for *M. gallisepticum* infection found, though not necessarily in the same individual (Table 1). Mycoplasma cultures were only successful from house finch samples yielding *M. gallisepticum.*

**Table 1:** Number of individuals testing positive for *M. gallisepticum* out of total number sampled by polymerase chain reaction (DNA detected  =  current infection) or by rapid plate agglutination (antibodies detected  =  previous infection).

			*M. gallisepticum* DNA detected					*M. gallisepticum* antibodies present				
Species/Family	Feeder use	Status	This study	Farmer et al (2005)	Luttrell et al. (2001)	Hartup et al. (2000)	sum	This study	Farmer et al (2005)	Luttrell et al. (2001)	Hartup et al. (2000)	sum
Downy Woodpecker (*Picoides pubescens*)	F	YR	**1/36**	-	-	-	1/36	**1/36**	-	-	-	1/36
Eastern Tufted Titmouse (*Baeolophus bicolor*)	F	YR	**0/36**	-	12/28	-	12/64	**5/36**	4/17	32/44	4/8	45/105
Black-capped Chickadee (*Poecile atricapilla*)	F	YR	**0/160**	-	-	0/2	0/162	**11/160**	-	-	-	11/160
White-breasted Nuthatch (*Sitta carolinensis*)	F	YR	**0/19**	-	-	-	0/19	**0/19**	-	-	-	0/19
American Robin (*Turdus migratorius*)	NF	SUM	**0/19**	-	0/3	-	0/22	**3/19**	0/2	10/16	-	13/37
Gray Catbird (*Dumetella carolinensis*)	NF	SUM	**0/45**	-	-	-	0/45	**3/45**	2/2	-	-	5/47
Cedar Waxwing (*Bombycilla garrulus*)	NF	YR	**1/10**	-	-	-	1/10	**0/10**	-	-	-	0/10
Common Yellowthroat (*Geothlypis trichas*)	NF	SUM	**1/13**	-	-	-	1/13	**0/13**	-	-	-	0/13
Northern Cardinal (*Cardinalis cardinalis*)	F	YR	**1/28**	-	0/6	-	1/34	**3/28**	5/49	33/157	-	41/234
American Tree Sparrow *(Spizella arborea*)	F	WIN	**1/46**	-	-	0/2	1/48	**2/46**	-	-	0/15	2/61
White-crowned Sparrow (*Zonotrichia leucophrys*)	F	WIN	**1/23**	-	-	-	1/23	**1/23**	-	-	-	1/23
White-throated Sparrow (*Zonotrichia albicollis*)	F	WIN	**1/21**		0/3		1/24	**1/21**	0/27	11/91	0/10	12/149
Song Sparrow *(Melospiza melodia*)	F	SUM	**1/121**	-	-	-	1/121	**7/121**	0/3	1/58	0/1	8/183
Dark-eyed Junco (*Junco hyemalis*)	F	WIN	**1/15**	-	-	-	1/15	**0/15**	-	0/37	0/5	0/57
Brown-headed Cowbird (*Molothrus ater*)	F	SUM	**0/11**	-	0/3	-	0/14	**1/11**	1/7	14/19	2/6	18/43
Red-winged Blackbird (*Agelaius phoeniceus*)	F	SUM	**3/74**	-	-	-	3/74	**0/74**	1/1	0/2	-	1/77
Purple Finch (*Haemorhous purpureus*)	F	YR	**1/28**	-	-	1/5	2/33	**0/28**	-	0/21	3/24	3/73
House Finch (*Haemorhous s mexicanus*)	F	YR	**40/331**		6/84	17/194	63/609	**11/331**	-	30/112	4/23	45/466
Pine Siskin (*Carduelis pinus*)	F	WIN	**2/154**	-	-	-	2/154	**3/154**	-	0/1	4/23	7/178
American Goldfinch (*Spinus tristis*)	F	YR	**15/537**	-	-	0/53	15/590	**8/537**	6/41	3/97	1/9	18/684
House Sparrow *(Passer domesticus)*	F	YR	**1/111**				1/111	**6/111**	6/33	0/24		12/168

Only species with 10+ individuals in Ithaca included; complete list in Table S1. -  =  no data. Feeder use: NF: species not using feeders; F: Status: YR: present year round; SUM: summer visitor; WIN: winter visitor.

### Variation between and within families

For eight families more than 40 individuals were sampled (Table 2). These families differed significantly in the proportion of individuals in which *M. gallisepticum* DNA was detected using PCR (0% to 5.5%; χ^2^ = 23.24, df = 7, P = 0.0015). If we remove house finches from the analysis differences between families are no longer statistically significant (χ^2^ = 7.29, df = 7, P = 0.40). House finches, therefore, rather than fringillids in general, more frequently carry *M. gallisepticum* DNA in their conjunctiva compared to other species (eight families without house finch: mean 1.68% ± SE 0.42; 95% confidence interval 0.69%–2.66%; compared to 12.1% in house finches; t = 4.02, df = 7, P = 0.005).

**Table 2:** Percentages of individuals trapped in Tompkins County that tested positive for *M. gallisepticum.*

Family or group	n	% PCR+	%RPA+	χ^2^(1df)	P
Picidae	41	2.4	2.4	0.00	1.0
Paridae	196	0.0	8.2	15.40	0.0001
Mimidae	46	0.0	6.5	3.10	0.08
Cardinalidae	42	2.4	9.5	1.91	0.17
Emberizidae	243	2.1	4.5	2.33	0.13
Icteridae	100	3.0	2.0	0.21	0.65
Fringillidae	1056	5.5	2.1	16.84	<0.0001
*without house finches*	725	2.5	1.5	1.72	0.19
*house finch only*	331	12.1	3.3	17.87	<0.0001
Passeridae	111	1.0	5.4	3.69	0.055

*% PCR+*: % of birds whose eye swab contained *M. gallisepticum* DNA; % RPA+: % of birds in which we detected *M. gallisepticum*-specific antibodies in a blood sample. The χ^2^ values and associated P-values stem from the comparisons of the number of individuals that were PCR or RPA positive.

The proportion of individuals in which *M. gallisepticum* antibodies were found using RPA varied significantly among families (2.0% to 9.5%; χ^2^ = 27.08, df = 7, P = 0.0003). The percentage of fringillids that were RPA positive is the second smallest (Table 2). After removing house finches the percentages varied between 1.5% (fringillids) and 9.5% (cardinalidae) with the differences between families still being statistically significant (χ^2^ = 29.36, df = 7, P = 0.0001).

Among the three fringillids with samples >30 individuals there exist clear differences in detections of *M. gallisepticum* DNA between house finches and other finches: *M. gallisepticum* DNA was found significantly more frequently in house finch samples (12.1%), than in samples taken from American goldfinches (2.8%; χ^2^ = 29.27, df = 1, P<0.0001) or from pine siskins (1.3%; χ^2^ = 15.46, df = 1, P = 0.0001), but house finches were not more likely to have antibodies than other finches (house finch versus American goldfinch χ^2^ = 3.22, df = 1, P = 0.07; house finch versus pine siskin *Carduelis pinus* χ^2^ = 0.71, df = 1, P = 0.30).

The first hypothesis, that *M. gallisepticum* would be more prevalent in fringillids than in other bird families is not supported if one excludes house finches. *M. gallisepticum* DNA is more often detected in house finches than in other bird species. Fringillids in general and house finches in particular, do not more frequently have detectable antibodies against *M. gallisepticum* compared to other bird species.

### Feeder use and *M. gallisepticum* exposure

If pathogen transmission is increased by using feeders one would expect that species associated with feeders would be more frequently exposed to *M. gallisepticum* compared to other species. To test that hypothesis we compared detections of *M. gallisepticum* in four species that do not use feeders (NF in Table 1) with 16 species that frequently use feeders (F in Table 1). We limited the comparison to species for which we had sampled at least 10 individuals. Because infection rates in house finches differ strongly from that in any other species (see above) we did not include house finches in these comparisons. Surprisingly the proportions of individuals in which we detected evidence of *M. gallisepticum* infection did not differ between the two groups. *M. gallisepticum* DNA was detected on average in 2.36% ± 0.51 (range: 0%–6.7%) of individuals in 16 species of feeder birds, and in 4.42% ± 2.60 of individuals (range: 0%–10%) in four species that do not come to feeders (Mann-Whitney U-test, corrected for ties P = 0.74). *M. gallisepticum* antibodies were detected on average in 4.47% ± 1.03 (range: 0%–13.9%) of 16 species using feeders compared to 5.62% ± 3.74 (range: 0%–15.8%) in four species not using feeders (Mann-Whitney U-test, corrected for ties P = 0.96). Association with feeders, therefore, does not explain *M. gallisepticum* prevalence.

### Migration status and *M. gallisepticum* prevalence

Given that mycoplasmal conjunctivitis varies seasonally in house finches, with minimal prevalence during the breeding season, and maxima during the winter months [26], [35], [36] one could expect that birds spending winter or parts of winter in Upstate New York would be more frequently exposed to *M. gallisepticum* compared to species that are summer visitors only. To test this hypothesis we compared the percentage positive (by PCR or RPA) for four winter visitors to six summer visitors (Table 1). Prevalence did not differ between the two groups based on detections by either PCR (U-test: P = 0.16) or RPA (P = 0.33).

### Prevalence and method of detection

Antibodies can be maintained in some house finches for more than a year, and long after the infection has been cleared from the conjunctiva [27]. If other species respond in a similar way to infection with *M. gallisepticum* one would expect that the proportion of individuals with antibodies (RPA-positive) would be higher than the proportion of individuals in which *M gallisepticum* DNA is detected (PCR-positive). In five families in this study the percentage of individuals that was RPA-positive was higher than the percentage that was PCR-positive. In only one family (Paridae) was this difference statistically significant (Table 2). In Picidae and in Icteridae the percentages were very similar. In Fringillidae, however, a significantly higher proportion of individuals were PCR-positive (5.5%) than RPA-positive (2.1%) (Table2). The difference was considerably larger among house finches (12.1% by PCR; 3.3% by RPA) than among other fringillids (2.5% by PCR versus 1.5% by RPA).

The method used, thus, does influence detection of exposure to *M. gallisepticum*, but not in a consistent way across bird species.

## Discussion

### Taxonomy and infection

All fifteen bird species (belonging to eight different families) for which we sampled at least 20 individuals were positive for evidence of *M. gallisepticum* infection. In 80% of them *M. gallisepticum* DNA was detected using PCR, while in the other three only antibodies were found. In one of the latter, the tufted titmouse, another study did detect *M. gallisepticum* using PCR, but culture was unsuccessful [18]. In that study, as in ours, the proportion of individuals in which antibodies were detected was much higher than the proportion of individuals positive by PCR [18]. Combining our data with results from other studies shows that a wide range of avian species are exposed to *M. gallisepticum* (Table S1): excluding poultry (Galliformes) species belonging to 18 families in three orders (Columbiformes, Piciformes and Passeriformes) have been documented to become infected with *M. gallisepticum*. Combining our study with that of Farmer et al. [19], Luttrell et al. [18] and Hartup et al. [17] *M. gallisepticum* DNA was detected in four fringillid species and 18 non-fringillid species, and in a fifth fringillid in Quebec [16]. One can only infer that in wild birds in NE North America (and likely elsewhere) exposure to *M. gallisepticum* must be widespread.

Excluding house finches, the proportion of individuals in which *M. gallisepticum* DNA is detected does not differ between families. This suggests that exposure to *M. gallisepticum* has a wide host range, but that house finches are uniquely susceptible to the current circulating strain in terms of active infection, clinical disease, and reservoir hosts capable of transmission. Among fringillids the proportion of individuals with *M. gallisepticum* DNA is about five times higher among house finches than among individuals of other species. Nevertheless, the proportion of birds in which we detected antibodies did not differ between fringillids. There are two possible non-exclusive causes for this. One is that a higher proportion of house finches become infected with *M. gallisepticum*. Another is that there is no difference in infection rates of different species, but because a *M. gallisepticum* infection causes severe and extended disease in house finches and does not in other species, *M. gallisepticum* DNA can be more easily detected in house finch conjunctival swabs than in other fringillids. Experimental infection studies [19], [30], [37] support both possibilities to some extent. We found that house finches sometimes become chronically infected with *M. gallisepticum* [38] while this has not been reported from other species. A chronic infection would increase the probability that *M. gallisepticum* DNA is detected. On the other hand *M. gallisepticum* within-group transmission is more successful among house finches than among American goldfinches, and transmission from house finches to conspecifics is more successful than from house finches to American goldfinches [37]. Both occasional chronic infections and differences in transmission rates between species would lead to higher proportions of house finches in which evidence of *M. gallisepticum* infection is found. One could speculate that over time, and because of repeated exposures to multiple strains (probably from poultry reservoirs), a *M. gallisepticum* strain evolved to become a successful pathogen of house finches.

### Feeder use and infection

Excluding finches, the data available indicate that birds using feeders and species that do not come to feeders had a similar probability of infection, a surprising result. This suggests that bird feeders are not the main source of infection, although they do play a role in transmission in house finches [39]. One can only wonder where species like the wood thrush or the common yellowthroat *Geothlypis trichas* get infected with *M. gallisepticum*. One could speculate that, with the exception of the known ‘house finch strain’ of *M. gallisepticum* (9, 11) multiple strains of *M. gallisepticum* could be responsible for the broader host species diversity described herein. *M. gallisepticum* strain diversity in the broader host range of wild birds could originate from contact with poultry (as per house finches), in particular ‘backyard poultry’, which are the most common reservoirs of *M. gallisepticum* and with little or no biosecurity to prevent contact with wild birds [40]–[42].

### Migration and infection

Given that disease prevalence in house finches fluctuates strongly between seasons, with very low prevalence during the breeding season, we expected summer resident species to be less exposed to *M. gallisepticum* than winter visitors. The small sample available does not suggest that such a difference would exist. Note that none of the summer-resident species for which we have data are truly long-distance migrants. Given that disease prevalence in house finches is higher in the southern USA [26] it cannot be excluded that these migrants became infected on their wintering grounds or during migration.

### Detection method and infection prevalence

Our data indirectly suggest that the course of infection varies among species, a result that is consistent with our finding that *M. gallisepticum* house finch strains do not cause disease in all experimentally infected species [19], [30], [43]. Specifically, the percentage of individuals in which *M. gallisepticum* DNA is detected by PCR or antibodies are detected by RPA varied strongly between families (Table 2). In two families (Picidae, Icteridae) the percentages were the same. In five families (Paridae, Mimidae, Cardinalidae, Emberizidae, and Passeridae) the percentages of individuals testing positive for *M. gallisepticum* by PCR were considerably smaller than those in which we detected antibodies.

The difference was especially large in the Paridae with antibodies in 8.2% of birds trapped, while no *M. gallisepticum* DNA was detected in any of the 196 individuals sampled by us. This is in contrast to Luttrell et al. 's [18] results who detected *M. gallisepticum* DNA by PCR in 43% of 28 seropositive tufted titmice in northeast Georgia. A possible reason for these different results is that we only sampled the conjunctiva, while Luttrell et al. [18] also sampled the sinus and trachea of euthanized seropositive birds. An alternate reason could be that *M. gallisepticum* does not grow well in the conjunctiva of Tufted Titmice (and other Paridae), but spreads to other parts of the body, so that antibodies continue to be produced. One must also include the possibility that there may be multiple strains of *M. gallisepticum* infecting wild bird populations. Experimental infection of black-capped chickadees showed that birds inoculated with a house finch strain of *M. gallisepticum* were successfully infected (*M. gallisepticum* recovered from conjunctival swabs, and antibodies detected) but did not develop clinical signs or visible eye lesions[43].

That the probability to find evidence of *M. gallisepticum* exposure increases with sample size in diverse host species may suggest the possibility of false positive test results. The performance characteristics of PCR and RPA tests for *M. gallisepticum* have been validated repeatedly in house finches, but to address the concern of false-positives in non-fringillids we experimentally inoculated black-capped chickadees and monitored them with the same tests used in house finches. We calculated that the probability of false positive RPA results in black-capped chickadees was at most 3.2% while the probability of false negative RPA results was at least 32% [43]. We can therefore conclude that the percentages of individuals testing positive for antibodies by RPA is probably an underestimate of the proportion of individuals that have been infected by *M. gallisepticum* and, therefore, false positive test results do not drive our results. On the contrary, the high proportion of RPA false negatives could result in underestimating the proportion of wild birds that become infected by *M. gallisepticum* if this is the only diagnostic method used.

Mycoplasma culture attempts are optimized when there are minimal delays from sample collection to direct inoculation of mycoplasma growth medium (e.g. FMS), followed immediately by incubation at 37° and daily observations for evidence of growth, all conducted by an experienced mycoplasma laboratory. Optimal conditions cannot be met for sample collection by cooperators of varied experience and from multiple locations, and is further complicated by the resulting need for sample storage and shipment to the mycoplasma laboratory. Faced with the challenges and costs (mainly over-night shipment) of sample collection for mycoplasma culture from remote locations over long time spans, we have tried various protocols as reflected herein. Our current mycoplasma sampling and culture protocol relies on conjunctival swabs inoculated to mycoplasma transport media (BD/Copan UTM; or Remel M4 or M5) followed by immediate storage at 4°C and overnight shipment on cold-packs to the mycoplasma laboratory where culture in FMS at 37°C is initiated immediately upon arrival. Even with this protocol, mycoplasma culture positive rates are highly variable among samples submitted, but overall about 50%. Collection of samples and mycoplasma isolates from wild birds at various locations is challenging and expensive, but a vital necessity to the ongoing and future study of mycoplasma conjunctivitis.

## Conclusions

Accumulating evidence suggests that the ecology of *M. gallisepticum* wild bird hosts is surprisingly diverse and includes birds that often come to feeders as well as species that never do, includes resident birds as well as migrants, and includes species from at least four orders.

A phylogenetic analysis of *M. gallisepticum* isolates (recovered largely from house finches, similar species, and poultry) indicates that in North America a single clade of the bacterium successfully jumped from poultry to house finches [11] with some transmission to similar species. Infections of the broader host range of wild birds could represent further host switching by the house finch clade of *M. gallisepticum,* but one must include the possibility that multiple lineages of *M. gallisepticum* are circulating in wild birds in North America and perhaps elsewhere. Additional work is needed to identify the phylogenetic relationships of the *M. gallisepticum* strain(s) infecting the entire array of wild bird species carrying this pathogen.

Furthermore, a geographically broader investigation is needed and with greater emphasis on *Mycoplasma* culture and isolation in order to establish whether our findings from the northeastern United States are representative, or whether there are systematic patterns of geographical variation in the extent to which *M. gallisepticum* is a multi-host pathogen. The few studies that have tested for the presence of *M. gallisepticum* in free-living birds in other parts of the world found evidence for its presence in wild house sparrows in India [21], wild tree sparrows in Japan [22] and in various corvid species in Scotland [23], [24] and in Malaysia [25]. One might speculate that *M. gallisepticum* could be widespread geographically and occur, usually without causing obvious clinical signs, in a wide variety of host species. The origin of *M. gallisepticum* strains that cause infections in wild birds in Europe and Asia clearly cannot be the same as that which caused the epidemic in North American house finches, because this strain only recently jumped from poultry to house finches [12], [13] and is geographically distant.

Finally, the route of infection for species that do not frequent bird feeders needs to be identified. The presence of *M. gallisepticum* infections in these non-feeder species suggests the possibility that *M. gallisepticum* can infect and persist in other species and is not merely the result of spill-over infections from house finches, contrary to the suggestion of Hartup [20]. It is possible that *M. gallisepticum* detected in wild birds around the globe represent past or recent introductions from ‘backyard poultry’, the most likely reservoirs of diverse *M. gallisepticum* strains, which are then maintained in those populations. Mycoplasmas are far more often commensals than pathogens, and as such often subclinical and chronic or latent, coupled with difficult to culture, isolate and identify: their role as infectious organisms has been controversial and underappreciated. Evidence for a diverse wild bird host range infected with *M. gallisepticum* may be another example of transmissible subclinical mycoplasmosis, achievement of an ideal host/parasite relationship. The emergence of a pathogenic *M. gallisepticum* strain in house finches may be the exception that has allowed us to identify the broader picture.

## Supporting Information

Table S1 Number of individuals testing positive for *M. gallisepticum* out of total number sampled by polymerase chain reaction (DNA detected  =  current infection) or by rapid plate agglutination (antibodies detected  =  previous infection) in this study (This study) and as reported by previous studies as indicated.
(DOCX)
