# Supplementary material for: Diverse Wild Bird Host Range of Mycoplasma gallisepticum in Eastern North America
Source: PLoS One. 2014 Jul 25;9(7):e103553. doi: 10.1371/journal.pone.0103553 (PMC4111589; doi:10.1371/journal.pone.0103553)
Supplement: Table S1 — Number of individuals testing positive for M. gallisepticum out of total number sampled by polymerase chain reaction (DNA detected = current infection) or by rapid plate agglutination (antibodies detected = previous infection) in this study (This study) and as reported by previous studies as indicated. (DOCX) [file pone.0103553.s001.docx]

Table S1. Appendix - Table 1. Number of individuals testing positive for *M. gallisepticum* out of total number sampled by polymerase chain reaction (DNA detected = current infection) or by rapid plate agglutination (antibodies detected = previous infection) in this study (This study) and as reported by previous studies as indicated.

|  | *M. gallisepticum* DNA detected | | | | | *M. gallisepticum* antibodies present | | | | |
| --- | --- | --- | --- | --- | --- | --- | --- | --- | --- | --- |
| Species | **This study** | Farmer et al (2005) | Luttrell et al. (2001) | Hartup et al. (2000) | sum | **This study** | Farmer et al (2005) | Luttrell et al. (2001) | Hartup et al. (2000) | sum |
| Mourning Dove (*Zenaida macroura* ) | **0/3** | 3/54 | - | - | 3/57 | **0/3** | 3/54 | 0/4 | - | 3/61 |
| Eurazian Collared Dove (*Streptopelia decaocto)* | - | - | - | - | - | **-** | 0/1 | - | - | 0/1 |
| **Columbidae** |  |  |  |  | **3/57** |  |  |  |  | **3/62** |
|  |  |  |  |  |  |  |  |  |  |  |
| Red-bellied Woodpecker *(Melanerpes carolinus)* | - | - | - | - | - | **-** | - | 0/6 | - | 0/6 |
| Yellow-bellied Sapsucker *(Sphyrapicus varius)* | **0/1** | - | - | - | 0/1 | **0/1** | - | - | - | 0/1 |
| Downy Woodpecker (*Picoides pubescens*) | **1/36** | - | - | - | 1/36 | **1/36** | - | - | - | 1/36 |
| Hairy Woodpecker *(Picoides villosus)* | **0/4** | - | - | - | 0/4 | **0/4** | - | 0/1 | - | 0/5 |
| **Picidae** |  |  |  |  | **1/41** |  |  |  |  | **1/48** |
|  |  |  |  |  |  |  |  |  |  |  |
| Eastern Wood Peewee (*Contopus virens*) | **0/1** | - | - | - | 0/1 | **0/1** | - | - | - | 0/1 |
| Traill's Flycatcher (*Empidonax traillii*) | **1/6** | - | - | - | 1/6 | **0/6** | - | - | - | 0/6 |
| Acadian Flycatcher (Empidonax virescens) | **0/2** | - | - | - | 0/2 | **0/2** | - | - | - | 0/2 |
| Least Flycatcher (*Empidonax minimu*) | **0/2** | - | - | - | 0/2 | **0/2** | - | - | - | 0/2 |
| Willow Flycatcher (*Empidonax traillii*) | **0/1** | - | - | - | 0/1 | **0/1** | - | - | - | 0/1 |
| Eastern Phoebe (*Sayornis phoebe*) | **1/3** | - | - | - | 1/3 | **0/3** | - | 0/9 | - | 0/12 |
| **Tyrannidae** |  |  |  |  | **2/15** |  |  |  |  | **0/15** |
|  |  |  |  |  |  |  |  |  |  |  |
| Red-eyed Vireo (*Vireo olivaceus*) | **1/3** | - | - | - | 1/3 | **0/3** | - | - | - | 0/3 |
| Yellow-throated Vireo (*Vireo flavifrons*) | **0/1** | - | - | - | 0/1 | **0/1** | - | - | - | 0/1 |
| **Vireonidae** |  |  |  |  | **1/4** |  |  |  |  | **0/4** |
|  |  |  |  |  |  |  |  |  |  |  |
| Blue Jay (*Cyanocitta cristata*) | **0/9** | - | - | - | 0/9 | **0/9** | 1/3 | 0/5 |  | 1/17 |
| **Corvidae** |  |  |  |  | **0/9** |  |  |  |  | **1/21** |
|  |  |  |  |  |  |  |  |  |  |  |
| Tree Swallow(*Tachycineta bicolor*) | **0/1** | - | - | - | 0/1 | **0/1** | - | - | - | 0/1 |
| **Hirundinidae** |  |  |  |  | **0/1** |  |  |  |  | **0/1** |
|  |  |  |  |  |  |  |  |  |  |  |
| Eastern Tufted Titmouse (*Baeolophus bicolor*) | **0/36** | - | 12/28 | - | 12/64 | **5/36** | 4/17 | 32/44 | 4/8 | 45/105 |
| Black-capped Chickadee (*Poecile atricapilla*) | **0/160** | - | - | 0/2 | 0/162 | **11/160** | - | - | - | 11/160 |
| Carolina Chickadee (*Poecile carolinensis*) | - | - | 0/1 | - | 0/1 | **-** | 2/17 | 2/14 | - | 4/31 |
| **Paridae** |  |  |  |  | **12/227** |  |  |  |  | **60/296** |
|  |  |  |  |  |  |  |  |  |  |  |
| Red-breasted Nuthatch (*Sitta canadensis*) | **1/2** | - | - | - | 1/2 | **0/2** | - | - | - | 0/2 |
| Brown-headed Nuthatch (*Sitta pusilla*) | - | - | - | - | - | **-** | - | 0/3 | - | 0/3 |
| White-breated Nuthatch (*Sitta carolinensis*) | **0/19** | - | - | - | 0/19 | **0/19** | - | - | - | 0/19 |
| **Sittidae** |  |  |  |  | **1/21** |  |  |  |  | **0/24** |
|  |  |  |  |  |  |  |  |  |  |  |
| Carolina Wren (*Thryothorus ludovicianus)* | - | - | - | - | - | **-** | 0/6 | 3/24 | - | 3/30 |
| House Wren (*Troglodytes aedon*) | - | - | - | - | - | **-** | 0/1 | - | - | 0/1 |
| Winter wren (*Troglodytes troglodytes)* | **0/1** | - | - | - | 0/1 | **0/1** | - | - | - | 0/1 |
| **Troglodytidae** |  |  |  |  | **0/1** |  |  |  |  | **3/32** |
|  |  |  |  |  |  |  |  |  |  |  |
| Ruby-crowned Kinglet (*Regulus calendula*) | **0/1** | - | - | - | 0/1 | **0/1** | 0/9 | 0/4 | - | 0/14 |
| Golden-crowned Kinglet (*Regulus satrapa*) | - | - | - | - | - | **-** | 0/5 | - | - | 0/5 |
| **Regulidae** |  |  |  |  | **0/1** |  |  |  |  | **0/19** |
|  |  |  |  |  |  |  |  |  |  |  |
| Eastern Bluebird (*Sialia sialis*) | **0/2** | - | - | - | 0/2 | **0/2** | - | 1/7 | - | 1/9 |
| American Robin (*Turdus migratorius*) | **0/19** | - | 0/3 | - | 0/22 | **3/19** | 0/2 | 10/16 | - | 13/37 |
| Wood Thrush (*Hylocichla mustelina*) | **1/5** | - | - | - | 1/5 | **4/5** | - | - | - | 4/5 |
| Hermit Thursh (*Catharus guttatus*) | - | - | - | - | - | **-** | 0/3 | 3/7 | - | 3/10 |
| **Turdidae** |  |  |  |  | **1/29** |  |  |  |  | **21/61** |
|  |  |  |  |  |  |  |  |  |  |  |
| Gray Catbird (Dumetella carolinensis) | **0/45** | - | - | - | 0/45 | **3/45** | 2/2 | - | - | 5/47 |
| Northern Mockingbird (*Mimus polyglottos*) | **-** | - | 0/1 | - | 0/1 | **-** | 3/15 | 2/17 | - | 5/32 |
| Brown Trasher (*Toxostoma rufum*) | **0/1** | - | - | - | 0/1 | **0/1** | 4/9 | 0/7 | - | 4/17 |
| **Mimidae** |  |  |  |  | **0/46** |  |  |  |  | **14/95** |
|  |  |  |  |  |  |  |  |  |  |  |
| Cedar Waxwing (*Bombycilla garrulus* ) | **1/10** | - | - | - | 1/10 | **0/10** | - | - | - | 0/10 |
| **Bombycilidae** |  |  |  |  | **1/10** |  |  |  |  | **0/10** |
|  |  |  |  |  |  |  |  |  |  |  |
| European Starling (*Sturnus vulgaris*) | **-** | - | 0/1 | - | 0/1 | **-** | - | 1/31 | - | 1/31 |
| **Sturnidae** |  |  |  |  | **0/1** |  |  |  |  | **1/31** |
|  |  |  |  |  |  |  |  |  |  |  |
| Yellow-rumped warbler (*Dendoica coronata)* | **-** | - | 0/1 | - | 0/1 | **-** | 0/27 | 9/21 | - | 9/48 |
| Pine Warbler (*Dendroica pinus*) |  |  |  |  |  | **-** | - | 1/1 | - | 1/1 |
| American Redstart (*Setophaga ruticilla*) | **0/1** | - | - | - | 0/1 | **0/1** | - | - | - | 0/1 |
| Black-and-white Warbler (*Mniotilta varia*) | **0/1** | - | - | - | 0/1 | **0/1** | - | - | - | 0/1 |
| Common Yellowthroat (*Geothlypis trichas*) | **1/13** | - | - | - | 1/13 | **0/13** | - | - | - | 0/13 |
| Yellow-breasted Chat (*Icteria virens*) | - | - | - | - | - | **-** | 0/2 | - | - | 0/2 |
| **Parulidae** |  |  |  |  | **1/16** |  |  |  |  | **10/66** |
|  |  |  |  |  |  |  |  |  |  |  |
| Northern Cardinal (*Cardinalis cardinalis*) | **1/28** | - | 0/6 | - | 1/34 | **3/28** | 5/49 | 33/157 | - | 41/234 |
| Rose-breasted Grosbeak (*Pheucticus ludovicianus*) | **0/9** | - | - | - | 0/9 | **1/9** | - | - | - | 1/9 |
| Indigo Bunting (*Passerina cyanea*) | **0/5** | - | - | - | 0/5 | **0/5** | - | - | - | 0/5 |
| Dicsissel (*Spiza americana*) | - | - | - | - | - | **-** | - | 0/1 | - | 0/1 |
| **Cardinalidae** |  |  |  |  | **1/48** |  |  |  |  | **42/249** |
|  |  |  |  |  |  |  |  |  |  |  |
| Eastern Towhee (*Pipilo erythrophthalmus*) | - | - | - | - | - | **-** | 0/7 | 0/7 | - | 0/14 |
| American Tree Sparrow *(Spizella arborea*) | **1/46** | - | - | 0/2 | 1/48 | **2/46** | - | - | 0/15 | 2/61 |
| Field Sparrow (*Spizella pusilla*) | **0/2** | - | - | - | 0/2 | **0/2** | - | 3/79 |  | 3/81 |
| Chipping Sparrow (*Spizella passerina)* | **0/6** | - | 0/1 | - | 0/7 | **0/6** | 4/20 | 4/88 | - | 8/114 |
| Savannah Sparrow (*Passerculus sandwichensis*) | **0/2** | - | - | - | 0/2 | **0/2** | - | 2/25 | - | 2/27 |
| White-crowned Sparrow (*Zonotrichia leucophrys*) | **1/23** | - | - | - | 1/23 | **1/23** | - | - | - | 1/23 |
| White-throated Sparrow (*Zonotrichia albicollis*) | **1/21** |  | 0/3 |  | 1/24 | **1/21** | 0/27 | 11/91 | 0/10 | 12/149 |
| Fox sparrow (*Passerella iliaca*) | **0/5** | - | - | - | 0/5 | **0/5** | - | 0/3 | - | 0/8 |
| Song Sparrow *(Melospiza melodia*) | **1/121** | - | - | - | 1/121 | **7/121** | 0/3 | 1/58 | 0/1 | 8/183 |
| Lincoln Sparrow (*Melospiza lincolnii*) | **0/1** | - | - | - | 0/1 | **0/1** | - | - | - | 0/1 |
| Swamp Sparrow (*Melospiza georgiana*) | **0/1** | - | - | - | 0/1 | **0/1** | 0/1 | - | - | 0/2 |
| Dark-eyed Junco (*Junco hyemalis*) | **1/15** | - | - | - | 1/15 | **0/15** | - | 0/37 | 0/5 | 0/57 |
| **Emberizidae** |  |  |  |  | **5/249** |  |  |  |  | **36/720** |
|  |  |  |  |  |  |  |  |  |  |  |
| Brown-headed Cowbird (*Molothrus ater*) | **0/11** | - | 0/3 | - | 0/14 | **1/11** | 1/7 | 14/19 | 2/6 | 18/43 |
| Red-winged Blackbird (*Agelaius phoeniceus*) | **3/74** | - | - | - | 3/74 | **0/74** | 1/1 | 0/2 | - | 1/77 |
| Common Grackle (*Quiscalus quiscula*) | **0/6** |  | 0/3 |  | 0/9 | **0/6** | - | 8/15 | - | 8/21 |
| Northern Oriole (*Icterus galbula*) | **0/9** | - | - | - | 0/9 | **1/9** | - | - | - | 1/9 |
| **Icteridae** |  |  |  |  | **3/106** |  |  |  |  | **28/150** |
|  |  |  |  |  |  |  |  |  |  |  |
| Purple Finch (*Haemorhous purpureus*) | **1/28** | - | - | 1/5 | 2/33 | **0/28** | - | 0/21 | 3/24 | 3/73 |
| House Finch (*Haemorhous s mexicanus*) | **40/331** |  | 6/84 | 17/194 | 63/609 | **11/331** | - | 30/112 | 4/23 | 45/466 |
| Common redpoll (*Carduelis flammea*) | **0/6** | - | - | - | 0/6 | **0/6** | - | - | - | 0/6 |
| Pine Siskin (*Carduelis pinus*) | **2/154** | - | - | - | 2/154 | **3/154** | - | 0/1 | 4/23 | 7/178 |
| American Goldfinch (*Spinus tristis*) | **15/537** | - | - | 0/53 | 15/590 | **8/537** | 6/41 | 3/97 | 1/9 | 18/684 |
| **Fringillidae** |  |  |  |  | **82/1392** |  |  |  |  | **73/1424** |
|  |  |  |  |  |  |  |  |  |  |  |
| House Sparrow *(Passer domesticus)* | **1/111** |  |  |  | 1/111 | **6/111** | 6/33 | 0/24 |  | 12/168 |
| **Passeridae** |  |  |  |  | **1/111** |  |  |  |  | **12/168** |

-
